# Family Climate and Intention to Use Cannabis as Predictors of Cannabis Use and Cannabis-Related Problems among Young University Students

**DOI:** 10.3390/ijerph18179308

**Published:** 2021-09-03

**Authors:** Olga Hernández-Serrano, Maria Eugènia Gras, Mariano Gacto, Alicia Brugarola, Sílvia Font-Mayolas

**Affiliations:** 1 Department of Psychology, Catholic University of Saint Anthony, Av. de los Jerónimos, 135, Guadalupe de Maciascoque, 30107 Murcia, Spain; ohernandez@ucam.edu; 2 Department of Psychology, Quality of Life Research Institute, University of Girona, Pujada de Sant Domènec, 9, 17004 Girona, Spain; eugenia.gras@udg.edu (M.E.G.); aliciabruga@gmail.com (A.B.); 3 Department of Physical Therapy, Faculty of Medicine, Campus Espinardo, University of Murcia, 30100 Murcia, Spain; marianogacto@um.es

**Keywords:** attitudes, cannabis use, cannabis-use problems, family climate, intention to use, self-efficacy, subjective norms, university students

## Abstract

Determining the predictive variables associated with cannabis use and cannabis-related problems can ease the identification of young cannabis consumers who can benefit from prevention interventions. This study aimed: (1) to describe, among university students, the cannabis use and cannabis-use problems, intention to use cannabis and family climate based on the gender and the people the student lives with; (2) to explore whether the family climate and intention to use cannabis are predictors of cannabis use and cannabis-related problems. The sample was composed of 339 Spanish undergraduates (51.9% females) in a 17-to-25 age range (19.67 ± 1.53). The variables were assessed through a battery based on the ESPAD survey, cannabis abuse screening test, cannabis use intention questionnaire and family climate scale. More men than women had used cannabis in the precedent year and showed greater intention to use cannabis, whereas more women than men showed greater self-efficacy in not using cannabis. The family climate did not predict cannabis use and cannabis-related problems. However, subjective norms and self-efficacy were key predictors of cannabis use and cannabis-use problems, respectively. Different factors seemed to predict the use cannabis in the past year versus cannabis-related problems, and these differences may help inform the development and delivery of preventative efforts.

## 1. Introduction

According to the latest European Drug Report [1], cannabis is the most widely used illegal drug. Cannabis use patterns range from occasional use to regular and dependent use. In Europe, 1% of adults in the general population use cannabis every day, and most of them (61%) are below 35 years of age. In this sense, the prevalence of cannabis use in the past year among young adults in a 15-to-24 year range is higher than among young adults between 15 and 34 years of age: 19.2% versus 15.4%, respectively. In addition, cannabis is the most commonly used illicit drug among university students [2]. Its potential detrimental effects on cognitive functions (i.e., attention, concentration, memory, verbal fluency, processing speed, planning, and decision making) are of particular concern [3,4,5], since cannabis use is also associated with academic performance (i.e., dropping out of college, poorer performance on exams, less time studying, lower class attendance, and the likelihood of earning a college degree) [6,7,8]. An important proportion of cannabis-using college students meet diagnostic criteria for disorder [3]. In Spain, the prevalence of problematic cannabis use in the population aged from 15 to 64 years was 22.5% during 2019/2020 [9]. More men than women had problematic cannabis use in the past year in Spain [9]. Given that the European prevalence of cannabis use is approximately five times higher than other substances [1], there is an urgent need to determine the predictive variables associated with cannabis use alongside the identification of young cannabis consumers who can benefit from selective preventative interventions.

Among the different models that try to explain cannabis use, the current study is based on the theory of planned behavior (TPB) developed by Ajzen [10]. The TPB suggests that the intention is formed from the attitude, the subjective norm (SN) and the perceived behavioral control (PBC). Attitudes have been defined as beliefs on the consequences of behavior [10], namely, the perception of the positive and negative consequences of using cannabis. The SN refers to the opinions existing in an individual’s social environment. for example, how people in the environment of the university student value the use of cannabis. The PBC refers to the easiness or difficulty that the person perceives about conducting the behavior. Self-efficacy refers to the belief that one can turn an idea or an intention into a successful achievement and that such an achievement can be fully explained by one’s own engagement and performance. The intention is considered as the main antecedent of the behavior and it is furthermore predicted from attitudes, the SN and PBC. This component establishes a direct relationship with behavior. In our particular case, the greater the intention to use cannabis, the higher the probability that cannabis will be used. Several studies have found that the variable intention significantly predicted cannabis use [11] and, in turn, intention was predicted based on attitudes and PBC, whereas SN did not have a decisive influence [12,13,14]. Along the same lines, other authors have demonstrated that PBC and attitude are predictive variables of the intention to use cannabis [15] and the intention of driving under the influence of cannabis [16]. A recent study has shown that the attitude towards marijuana use, subjective norms, and behavioral intention to use marijuana positively correlate with marijuana use among young Iranian adults. In contrast, higher self-efficacy is associated with lower marijuana use. However, the multiple regression analysis shows that solely a positive attitude to marijuana use, lower self-efficacy in resisting its use and higher behavioral intention predict actual cannabis use [11]. In contrast, Dempsey et al. [17] found that the perceptions of peers’ cannabis use were associated with personal cannabis use (odds ratio (OR) = 1.42; 95% CI (1.22, 1.64)) among a large sample of university students from Belgium, Denmark, Germany, the Slovak Republic, Spain, Turkey, and the United Kingdom. Despite the fact that there are multiple theoretical approaches in the field of drug addiction, the TPB has already been successfully used to identify predictors of marijuana use among adolescents and young adults [18,19,20].

Environmental factors, especially the family climate, are strongly associated with the onset of substance use [21,22]. One of the sources of stress commonly studied within the core of the family is the possibility of conflicts between or across family members: studies have found that living in families with high levels of conflict indirectly predicts drug use [23,24] and family conflict has also a significant influence on drug use [25]. Family expressiveness is another variable studied within the family; authors indicate that substance use is associated with poorer communication within the family [26,27]. Conversely, other authors suggest that interventions to promote family cohesion may have considerable benefits in reducing substance abuse among adolescents [28]. In this sense, family cohesion indirectly predicts substance use among adolescents (aged 12–18) with cannabis abuse or dependence [23]. In contrast, youth exposed to family cohesion are less likely to use substances [29]. Furthermore, when predicting drug use among secondary students, gender differences have been found, while these differences have not been stated among university students. For example, McLaughlin [30] investigated the link between the psychological and emotional characteristics involved in high-risk drug use among university and secondary school students: this study found that drug use among females was predicted by family expressiveness, whereas drug use among males was predicted by family conflict only in secondary students, but not among university students. It is important to determine which family factors could predict cannabis use and abuse since recent studies suggest that college students reporting substance use and family dysfunctionality are more likely to exhibit symptoms of depression, anxiety and stress [31].

Despite that cannabis is the most commonly used illicit drug among young adults [1] and university students [2,32], the vast majority of studies focusing on cannabis use and based on the theory of planned behavior have been conducted among adolescents [18,19,33]. Conversely, recent studies have found that different factors predict cannabis use versus problematic cannabis use, for instance, Mader et al. [34] found that religiosity and living with parents or relatives were both associated with lifetime cannabis use. However, male gender, earlier age of first cannabis use, cannabis use motives, past six-month medicinal use and increased frequency of deep inhalation during consumption were found to be correlates of problematic cannabis use among Canadian university students. In the same line, Lobato et al. [35] found that perceived past parental drug use increases the likelihood of adolescent cannabis use in general, but not its problematic use. In this sense, further research on the predictive factors of cannabis use versus problematic cannabis use based on the TPB and family environment is required among university students. Given this knowledge gap, this study aimed: (1) to describe, among university students, the cannabis use, cannabis-related problems, intention to use cannabis (SN, self-efficacy, attitudes, and intention to use), and family climate (cohesion, expressiveness, and conflict) based on the gender and the people the student lives with; (2) to explore whether the family climate (cohesion, expressiveness, and conflict) and intention to use cannabis (SN, self-efficacy, attitudes, and intention to use) are predictors of cannabis use and cannabis-use problems.

## 2. Materials and Methods

### 2.1. Participants

Participants were students in physical therapy from the University of Girona, Catalonia, Spain. The center and the sample of the study were selected by means of a nonprobability-purposive sampling method. In physical therapy, 362 were enrolled university students from first and second year of studies and 23 did not attend class on the day and time of data collection. The sample was entirely composed of students (100%) from compulsory-attendance classes from first and second year of studies, taught by lecturers collaborating in the current research. A total of 339 university students (female 176, 51.9%; male 163, 48.1%) in a 17-to-25 age range (19.67 ± 1.53) participated in the study. A total of 72 subjects (21.2%) lived with a family member and 267 (78.8%) lived alone, with friends or roommates, in a student residence or in other similar circumstances. Concerning cannabis-related problems (measured throughout the Cannabis Abuse Screening Test), a percentage of 4.2% (*n* = 14) of the sample showed scores equal or superior to nine, indicating cannabis-dependence, whereas 1.8% (*n* = 6) scored in a 7-to-8 range (indicating a moderate addiction), a total of 17.7% (*n* = 60) ranged between 1 and 6 (indicating cannabis use but not problematic use) and a percentage of 76.4% (*n* = 259) had never developed any of the six behaviors corresponding to the problematic consumption. The ethical code number of the study is CEBRU0003-2017 and the date of approval was 4 May 2017.

### 2.2. Measures

Sociodemographic variables (age, gender, and residence status) were assessed by ad-hoc questionnaires.

*Cannabis Use* (questionnaire based on the ESPAD survey, 2011) [36]. Questions on cannabis use in the past twelve months are included, with seven response options: 1 = *never*, 2 = *1–2 times*, 3 = *3–5 times*, 4 = *6–9 times*, 5 = *10–19 times*; 6 = *20–29 times*, 7 = *30 or more times*. Responses are categorized into two different categories (0 times, once or more).

*Cannabis Use Intention Questionnaire (CUIQ)* [37] was composed of a total of 22 items and four different subscales (attitude, subjective norm, self-efficacy, intention to use). The reliability coefficients for self-efficacy and intention to use exceeded 0.70 (0.79 and 0.77, respectively), whereas concerning the subscales attitude and subjective norm they were 0.68 and 0.58 respectively [37]. The four aforementioned subscales are developed and explained below:

*Attitude Scale*. This scale presents two dimensions: (a) four items in the first dimension measure the extent to which the use of marijuana or hashish can have an impact on several beliefs (for instance, “it helps you relax”), with a 5-point Likert scale, from 1 (not probable at all) to 5 (highly probable), and (b) a second dimension with four items measures to which extent each one of the aforementioned aspects is important (for instance, “relax: is it important to you?”), in a 5-point scale, from 1 (no important at all) to 5 (highly important). This second dimension is based on the assumption that attitudes do not specifically depend on beliefs, but on the assessment that each individual makes of each one of the aforementioned beliefs. Therefore, two subjects can believe with similar strength that cannabis may help him/her relax, but one of them can perceive relaxation as something extremely positive, whereas the other can consider this relaxation as less desirable. These two dimensions are combined in a multiplicative fashion in order to obtain an only score. 

*Subjective Norm Scale*. This scale presents two different dimensions: (a) normative beliefs concerning other peers (close friends, person he/she likes, and classmates) are operationalized by means of three items (for instance, “if I use marijuana or hashish: would my close friends agree with me?”) on a 1-to-5 Likert scale (1: strongly disagree; 5: strongly agree); and (b) motivation to conform to referents, by means of three items assessing these people’s opinion with respect to the use of marijuana or hashish (for instance, “the opinion of my friends: is it important for me?”), on a response scale from 1 (no important at all) to 5 (very important). These two dimensions are also combined in a multiplicative fashion in order to obtain a sole final score.

*Self-efficacy Scale*. The perceived behavioral control has been operationalized as a measure of a self-efficacy since both concepts correspond to the perceived ability to develop a specific behavior (Bandura, 1982). This scale comprises a set of beliefs on the self-ability to avoid cannabis use in different situations (for instance, being able to “stay with friends without smoking joints”). It encompasses five items, measured with a 5-point Likert scale, from 1 (unable) to 5 (completely able). 

*Intention to Use Scale*. It is composed of three items on “the intention to use marijuana or hashish in the near future”, “having planned to use marijuana or hashish in the near future” and “in case of having the opportunity, willing to use marijuana or hashish”. The response scale is 5-point Likert based, from 1 (definitely not) to 5 (definitely yes). 

*Cannabis Abuse Screening Test (CAST)* [38]. Screening scale to identify problematic cannabis was used. It is short, it was easy to administer, and validated in Spanish. It has shown adequate psychometric properties to evaluate the severity of the dependence to cannabis taking into account different components of validity among young people and young-adults [39,40]. It comprises six items, and it evaluates the frequency, within the prior twelve months, of the following events: recreative use (1. “Have you smoked cannabis before noon?”; 2. “Have you smoked cannabis when you were alone?”; 3. “Have you had memory troubles when smoking cannabis?”; 4. “Have your friends or your relatives asked you to decrease the use of cannabis?”), the failed attempts to quit (5. “Have you already tried to decrease or quit the use of cannabis without succeeding?”) and the troubles related to the use of cannabis (6. “Have you ever had troubles due to your cannabis consumption -arguments, quarrels, accidents, bad academic results, etc.-?”). Each item response corresponds to a Likert scale (0 “never”, 1 “rarely”, 2 “sometimes”, 3 “often”, and 4 “quite often”). In its complete version (CAST-f), the score is the addition of scores from each individual item, on a 0-to-24 range overall. The internal consistencies of the CAST (f) were good, with Cronbach’s alpha value of 0.75 [40]. The cut-off points used in the current study were 9 for dependence (following DSM-IV [41]) and 7 for moderate addiction (following DSM-V [42]) [39,40]. However, categories ‘7-8’ and ‘9 or more’ were grouped, due to the unfulfillment of the conditions to apply the test.

*Family Climate Scale (FCS)* [43] was adapted to Spanish by Seisdedos, de la Cruz, and Cordero [44]. The current study used solely the dimension “interpersonal relationships”, composed by 27 items encompassed in three subscales (cohesion, expressiveness and conflict) and 9 items in each subscale. This dimension assesses the sense of belonging to the family, the expression of feelings and conflicts. More specifically, the subscale “cohesion” corresponds to the degree of the perception on the family support and engagement that the adolescent has with respect to his/her family (for instance, “in my family we support and really help each other”). The subscale “expressiveness” reveals the perception that the adolescent has on the extent to which the members of his/her family openly express their feelings (for instance, “my family members keep their feelings for themselves”). The subscale “conflict” corresponds to the perception of the adolescent on the existence of conflicts between or across family members (for instance, “in my family we quarrel quite often”). The response options in this instrument are binary (true or false). Concerning the construct validity of the current instrument, this scale is adequate for identifying relevant characteristics concerning the psychological adaptation of the family members [45]. Its reliability indexes in previous studies has been satisfactory, ranging from 0.52 to 0.86 [46,47,48].

### 2.3. Procedure

The present study was approved by the Research Committee of the University of Girona (UdG). Prior to data collection, an interview with the managing staff of the university school was conducted, with the aim of expounding the main characteristics of the research and to request participation. The request for participation from students was conducted by means of specific written consent. All students voluntarily accepted participation voluntarily after being informed on the purpose of the study and the respect to the ethical principles in research. Participants provided answers to the questionnaires within lecture-rooms of the university during the second semester, in February 2019.

### 2.4. Statistical Analysis

Descriptive statistics were used to characterize the cohort at baseline, including sociodemographic characteristics and the variable “people the student lives with” (with or without people from the family environment). The arithmetic mean and standard deviation of quantitative variables and the percentage of qualitative variables were calculated.

The frequency of cannabis use (not consuming or consuming once or more times) and cannabis-related problems (dependence on cannabis, moderate addiction, non-problematic use, no behaviors associated with problematic use) based on gender and the variable “people the student lives with” (with or without people from the family environment) were analyzed using Pearson’s Chi-square test.

Next, bivariate analyses were conducted using independent t test to study the intention to use cannabis and family climate based on the gender and people with whom the student lives (with or without people from the family environment). Independent t test was also used to analyze the family climate based on cannabis use in the last 12 months (consumption/non-use).

Pearson’s correlation was used to study the relationship between family climate and problematic cannabis use and intention to use.

In addition, a binary stepwise logistic regression model was fitted to predict cannabis use in the last 12 months (criterion variable) based on family climate (subscales: cohesion, expressiveness, and conflict) and intention to cannabis use (subscales: attitudes, subjective norm, self-efficacy and intention to consume) controlling for age and gender.

Finally, multiple stepwise regression was used to predict the score on the cannabis-use problems (criterion variable) based on family climate and intention to use cannabis, controlling for age and gender.

All analyses were performed using the statistical package SPSS version 24.0, IBM Corp., Armonk, NY, USA.

## 3. Results

### 3.1. Cannabis Use, Cannabis-Use Problems, Intention to Use Cannabis and Family Climate Based on Gender and People the Student Lives with

#### 3.1.1. Cannabis Use in the Last Year Based on Gender and People the Student Lives with (Living or Not Living with Relatives)

Significantly more men than women had used cannabis once or more in the twelve months prior to the study. Despite the fact that there were less cannabis users among the participants living with family members compared to subjects not living with family members, differences were not statistically significant (see Table 1).

#### 3.1.2. Cannabis-Use Problems Based on Gender and People the Student Lives with (Living or Not Living with Relatives)

Table 2 shows the distribution of the aforementioned results by gender and residence status. Notwithstanding that more men than women and more young people not living with parents with respect to those living with them showed scores associated to a moderate addiction and to dependence, differences were not statistically significant. 

#### 3.1.3. Intention to Use Cannabis and Family Climate Based on Gender and People the Student Lives with (Living or Not Living with Relatives)

Statistically significant differences were found solely based on gender, but not on residence status. Women scored significantly higher than men on self-efficacy to not using cannabis and significantly lower on the close intention to use cannabis. Please, see Table 3.

### 3.2. Relationship between Family Climate, Cannabis Use and Cannabis-Use Problems, and Intention to Use Cannabis

#### 3.2.1. Family Climate Based on Cannabis Use in the Last Year

Non-users of cannabis obtained significantly higher mean scores on cohesion and expressiveness, whereas users scored higher on the conflict subscale (see Table 4). 

#### 3.2.2. Correlation between Family Climate, Cannabis-Use Problems, and Intention to Cannabis Use

Results from the Pearson correlation show that cannabis-use problems is inversely associated to family cohesion and directly to the conflict subscale, while the SN and intention to use are inversely associated to family cohesion. Moreover, future intention to use is associated to higher scores in the conflict subscale (see Table 5). 

### 3.3. Regression Models to Predict Cannabis Use and Cannabis-Use Problems

#### 3.3.1. Binary (Stepwise) Logistic Regression to Predict Cannabis Use in the Last Year

Table 6 shows the results from the binary stepwise logistic regression. Variables that best predicted cannabis use in the past twelve months were subscales “subjective norm” and “intention to use”. The model is well fitted (χ^2^_(4)_ = 173.53; *p* < 0.001; coefficient of determination Nagelkerke’ s *R*^2^ = 0.57), and the percentage of adequate classifications is 85.2% (93.3% of non-users and 70.4% of users). 

#### 3.3.2. Multiple Regression (Stepwise) to Predict Cannabis-Use Problems

The best predictors for problematic cannabis use were self-efficacy not to use and intention to use: the lower the self-efficacy and the higher the intention to use, the higher the score of cannabis-use problems was. The model explains a 46.6% of the variability and it is well adjusted (*R*^2^ = 0.46; *F* = 69.78; *p* < 0.001). Please, see Table 7.

## 4. Discussion

The first objective of the current study was to describe the frequency of cannabis use in the last year, problematic cannabis use, intention to use cannabis and family climate based on both gender and people with whom the student lives (with or without people from the family environment) among young university students.

In relation to the gender-based differences, more men than women used cannabis in the year prior to the study. This result is consistent with previous studies [1,49,50,51]. However, no gender differences were found on the CAST. Several explanations arise for the absence of differences between men and women: (1) the prevalence of problematic use has progressively decreased among men, the group with the highest prevalence at onset, from 3.5% in 2013 to 2.5% in 2017 whereas the problematic use among women has not experienced changes [1]; (2) according to the Spanish Observatory of Drugs and Drug Addiction [9], the highest percentage of problematic use corresponds to the 25-to-34 age group, and the age range of the participants in the present study encompassed the 17-to-25 year-range; (3) the combined use of cannabis and tobacco was not considered in the present study. Recent literature suggests that in Europe, unlike in America, the most common route of administration of tobacco is by mixing cannabis with tobacco in joint form [52]. Studies on the use of cannabis mixed with tobacco found that the percentages of non-dual consumers (cannabis with tobacco users and polydrug users, concurrent users: cannabis with tobacco; simultaneous users: cannabis with tobacco, and cannabis with tobacco with alcohol) did not significantly differ between male and female university students [53]. This result is consistent with previous research assessing polydrug use (consumers of cannabis plus alcohol and/or tobacco consumers) in university students since no differences by gender were found [50].

Focusing on the CUIQ subscale, more women than men showed higher levels of self-efficacy not to use cannabis while more men than women showed greater intention to use cannabis. These results are consistent with previous studies showing higher levels of self-efficacy among women [54]. On another note, the intention to use cannabis significantly predicted cannabis use (and cannabis-use problems) in the present study, in line with other studies [12,13,14,37]. The intention to use cannabis in our study was higher in men than in women, considering that the intention to use is a predictor of behaviors of cannabis consumption and the fact that the intention to use was higher in men than in women, this result is consistent with data from the last report on drugs [1], which reports that, among all the illegal drugs, cannabis is the most-consumed substance, with a maximal prevalence within the 15-to-24 age range and mainly used by men. In this sense, further research should consider protective factors in the use of cannabis within the framework of gender-tailored preventative programs.

When comparing students living with relatives and students who live with other people (e.g., friends or roommates in a student residence or in other circumstances), no differences were found based on the frequency of cannabis use during last year, the problematic cannabis use, intention to use cannabis and family climate. In contrast, other studies found a relationship between living alone and the risk of substance use [55] and living with parents or relatives and lower-lifetime cannabis use [34,56]. A feasible explanation for the results of the current study may be linked to the place of the residence rather than to the people with whom the student lives, namely, not living with relatives may imply living in a rented apartment, in a residence hall, student housing or in the home of a family different to the biological one. In this sense, Sánchez [57] found a significant relationship between cannabis use and living with a member of the family. However, when analyzing the residence status, a significant relationship was found between the fact of living in a rented apartment and the use of cannabis, but no relation was found when focusing on students living in a residence hall. Residence halls provide different academic, cultural, sports-related, academic competence-development, solidary and religious activities. In this sense, several environments as residence halls or student housings could exert a protective influence on the use of cannabis.

The main objective of this study was to examine whether the family climate and the intention to use cannabis could have determined the use of cannabis in the past year and the problematic cannabis use among university students. Our results show that the best predictors of cannabis use in the last year were SN and the intention to use cannabis. This result is consistent with previous research stating the association of perceived norms with more frequent marijuana use and drug-related consequences among university students [17,58,59]. In contrast, other studies did not conclude that SN was a determining variable [12,13,14,15,16]. Three possible explanations for our results therefore arise:

(1) A meta-analysis found that when the type of measure was used as a moderator, the poor performance of the subjective norm component was shown to be a function of measurement (multiple-item scale, single item, general social pressure multiplied by motivation to comply, normative beliefs as direct predictors of intention, social support and unspecified). This meta-analysis specifically highlights the measurement as its principal weakness, given that the majority of TPB studies have used single-item measures [60]. In the current study, a recently developed and validated scale was used with the aim of assessing the risk of cannabis use; unlike other different instruments (e.g., Olivar and Carrero [61]), the scale CUIQ exactly and precisely follows the considerations stated by Ajzen [10] measuring factors in a direct fashion [37]. In this sense, SN was assessed through the following pathways: (a) asking students about their perception of the degree of approval of a possible self-consumption of cannabis by their close friends, person that the student likes and classmates and (b) asking students how important the opinion of close friends, person he/she likes and colleagues is on the use cannabis. Clearly, this component requires further empirical attention;
(2) Authors have shown that specific variables could mediate in the effect between SN and the intention to use cannabis. For instance, Terry and colleagues [62] have shown that the identification with a behaviorally relevant group moderates the effects of group norm on the intention. Along the same lines, Neighbord et al. [63] found that injunctive norms (e.g., perceived approval of marijuana use by one’s peers) were significantly associated with cannabis use only in the presence of greater social expectancies. In this sense, further research is required about the variables that could mediate between subjective norms and cannabis use;
(3) A third feasible explanation could stem from the relationship between the frequency of cannabis use and the perception of cannabis use within the group of friends. In agreement with the last report on d rugs in Spain [9], the frequency of cannabis use was higher in students with friends (all or the vast majority) having used cannabis in the last month with respect to students having no cannabis users among their friends.

On another note, self-efficacy and intention to use were the best predictors of cannabis-use problems. This result is consistent with previous studies, finding a relationship between self-efficacy and problematic cannabis use through the CAST test [37]. Other studies found that self-efficacy was related to the frequency of marijuana use. Stephens, Wertz and Roffman [64] found that self-efficacy was a relatively strong predictor of the frequency of marijuana use. In a study comparing four treatment approaches for the dependence of marijuana [65,66], the duration of continuous abstinence over the course of a year was best predicted by self-efficacy at posttreatment, which was a better predictor than abstinence during the treatment period. However, a review found differences in the impact of self-efficacy depending on the particular outcome assessed, such that systematic studies employing different outcomes (e.g., abstinence, frequency of consuming, quantity consumed) and different outcome assessment durations would be valuable [67].

In relation to the family climate, participants not having used cannabis in the past twelve months got mean scores significantly higher in cohesion and expressiveness with respect to users, while users scored higher in the conflict scale with respect to non-users. Moreover, an indirect correlation was found between family cohesion with cannabis-use problems and the intention to use cannabis, and also a direct relationship between family conflict with cannabis-use problems and the intention to use cannabis. However, within the regression model, family climate was neither a predictor of cannabis use in the past year nor of cannabis-related problems. According to the aforementioned result, it is of paramount importance to highlight that around 80% of the sample lived with friends or flat-mates, in a student residence, alone or in other similar circumstances. In this sense, it is important to underline the characteristics of the university period and the changes that a young student faces during this phase of his/her life. The university sphere provides the student with a stage of personal development, expectations, professional projects and new challenges. Most of these students usually change their residence status and set themselves apart from their parental references. Within this context, the family core, which plays a referential key role during the adolescent period, decreases its influence [68]. As the young person grows up and achieves higher levels of independence, the influence of parents towards children loses strength [69]. In this sense, variables associated with the family (as, for instance, the family climate) are expected to show less influence. For example, authors found that support from the father negatively predicted drug use while support from adolescent’s couple predicted it in a positive way among adolescents from 15 to 17 years of age [70]. These same authors also found that the friend’s perceived social support was positively related to the existence of communication problems with the father [70]. This could be interpreted as a greater search for support in personal relationships outside the family when the student perceives communication problems with his/her parents. Other possible justification to the results stemming from the current study could be associated with the relationship between the use of substances and the influence of other variables associated with family (but different from the family climate). For instance, authors have found that less family monitoring, family support, and family self-esteem are associated with substance use [71,72,73]. Recently, the optimal parenting style is being investigated due to its relationship with other variables such as socialization [72,74,75,76,77]. In this sense, parenting style could still be an under-researched variable in relation to cannabis use. Finally, the family climate scale (FCS) used in the present study to assess family relationships has been used mainly in the adolescent population (up to 18 years old), but not in the university population. More research is required on the family and cannabis use in the university population.

Our findings should be interpreted in light of the study limitations: (1) the sample corresponds to a specific geographical region and area of knowledge or study (university students in health sciences), which hinders an eventual generalization to other contexts. Further studies with larger sample sizes and from different geographical areas should be conducted; (2) no temporary relationships have been explored, since the present study is cross-sectional. A longitudinal study observing changes over time would be required to complete this research; (3) given that the consumption of drugs may or may not be generally accepted by society, the answers given by the participants in the self-report could be inflated or deflated by a social desirability bias. The use of biological tests for assessing polydrug use and the inclusion of different informants (peers and/or parents) would provide more reliable measures of those variables. Nevertheless, confidentiality of responses was guaranteed in an effort to minimize this bias; (4) given that we have evaluated cannabis use in the last 12 months, it would be convenient for future studies to analyze whether cannabis use is conducted alone or in combination with tobacco. Recent literature suggests that in Europe, unlike in America, the most common route of administration of tobacco is by mixing cannabis with tobacco in joint form [52]. This form of consumption increases the probability of developing a greater dependence to cannabis [78], and in this sense, the predictive variables of cannabis use could be different; (5) a number of authors have argued that the way in which norms are conceptualized within the TPB framework fails to tap important facets of social influence (e.g., Conner and Armitage [79]; Terry, Hogg, and White [80]). In this sense, certain studies highlight the importance of distinguishing between two types of social norms [63]: descriptive norms (e.g., perceived frequency of marijuana use by one’s peers) and injunctive norms (e.g., perceived approval of marijuana use by one’s peers) because the results suggested that the perceptions of friends’ marijuana use were most strongly associated with marijuana use in comparison with perceived injunctive norms [63]. There is also evidence to support the inclusion of moral norms (e.g., Conner and Armitage [79]) within the TPB. Since this distinction has not been considered in the present study, this lack of distinction makes it difficult to compare our results with previous studies; (6) in the current study, we assess the use of cannabis in the past year as a criterion variable. Nevertheless, the variable “cannabis use” as criterion variable has been used, in previous studies, using frequencies of consumption with a wide variability (three past months, six past months, …). These differences related to the criterion variable hinder any possible comparison with other studies. Further research should consider the aforementioned variability; (7) finally, we have not considered the possibility that cannabis use or intent to cannabis use may be based on perception that such use could alleviate certain underlying physical or psychological distress [81]. In this sense, future studies should include self-report and disclosure of physical distress caused by accident or injury or other, as well as a self-report/disclosure of emotional distress or treatment for emotional distress.

## 5. Conclusions

In summary, more men than women used cannabis in the last year and showed greater intention to use cannabis, whereas more women than men showed higher levels of self-efficacy on non-using cannabis. Concerning the past year, different factors seem to predict cannabis use versus cannabis-use problems, and these differences may help inform the development and delivery of preventative efforts. Given that the best predictor for cannabis use was “SN”, and conversely, “self-efficacy not to use cannabis was the largest correlate of cannabis-use problems, education and preventative strategies tailored to the aforementioned variables may benefit students with cannabis use and problematic cannabis use.

## Figures and Tables

**Table 1:** Number and percentage of times students used cannabis in the past year based on gender and people the student lives with (living or not living with relatives).

Cannabis Use				
	Never	1 or More Times	χ^2^	*p*
Gender	% (*n*)	% (*n*)		
Male	59.5 (97)	40.6 (66)	4.17	0.041
Female	70.5 (124)	29.5 (52)		
Living with relatives			0.29	0.59
Yes	68.1 (49)	32.0 (23)		
No	64.4 (172)	35.5 (95)		

**Table 2:** Number and percentage of cannabis-use problems in the past year based on gender and people the student lives with (living or not living with relatives).

Cannabis-Use Problems					
	0	1–6	7 or More	χ^2^	*p*
Gender	% (*n*)	% (*n*)	% (*n*)		
Male	71.2 (116)	20.9 (34)	8.0 (13)	5.19	0.08
Female	81.3 (143)	14.8 (26)	3.9 (7)		
Living with relatives					
Yes	79.2 (57)	18.1 (13)	2.8 (2)	1.61	0.45
No	75.7 (202)	17.6 (47)	6.7 (18)		

**Table 3:** Mean and standard deviation of intention to use cannabis and family climate based on gender and people the student lives with (living or not living with relatives).

	CUIQ Attitudes	CUIQ SN	CUIQ Self-Efficacy	CUIQ Intention	FCS Cohesion	FCS Expressiveness	FCS Conflict
	M (SD)	M (SD)	M (SD)	M (SD)	M (SD)	M (SD)	M (SD)
Male	1.44 (1.0)	1.56 (0.9)	4.52 (1.0)	1.80 (1.2)	7.00 (1.8)	5.51 (1.7)	3.70 (1.7)
Female	1.27 (0.9)	1.56 (1.0)	4.86 (0.5)	1.36 (0.8)	7.22 (1.7)	5.81 (1.9)	3.35 (1.6)
*t*	1.60	0.03	−4.16	3.97	−1.16	−1.48	1.93
*(p)*	(0.11)	(0.98)	(<0.001)	(<0.001)	(0.25)	(0.14)	(0.054)
*d*	--	--	0.45	0.43	--	--	--
Living with relatives	1.52 (0.9)	1.59 (0.8)	4.74 (0.7)	1.51 (1.0)	6.82 (2.0)	5.55 (1.9)	3.55 (1.6)
Not living with relatives	1.31 (1.0)	1.55 (1.0)	4.69 (0.8)	1.59 (1.0)	7.20 (1.7)	5.69 (1.8)	3.51 (1.6)
*t*	1.69	0.35	0.45	−0.58	−1.63	−0.61	0.16
*(p)*	(0.09)	(0.73)	(0.66)	(0.57)	(0.10)	(0.54)	(0.87)

**Table 4:** Mean and standard deviation of family climate based on cannabis use in the last year.

	FCS Cohesion	FCS Expressiveness	FCS Conflict
Not cannabis use	7.29 (1.5)	5.83 (1.7)	3.36 (1.6)
Cannabis use	6.79 (2.0)	5.35 (1.9)	3.82 (1.7)
*t*	2.26	2.18	−2.43
*(p)*	(0.025)	(0.03)	(0.02)
*d*	0.29	0.27	0.28

**Table 5:** Correlation between family climate, cannabis-use problems, and intention to cannabis use.

	CAST	CUIQ Attitudes	CUIQ SN	CUIQ Self-Efficacy	CUIQ Intention
	*r* (*p*)	*r* (*p*)	*r* (*p*)	*r* (*p*)	*r* (*p*)
Cohesion	−0.12 (0.03)	−0.11 (0.051)	−0.12 (0.03)	0.08 (0.11)	−0.16 (0.003)
Expressiveness	−0.02 (0.75)	−0.07 (0.20)	−0.03 (0.58)	0.05 (0.33)	−0.09 (0.10)
Conflict	0.19 (<0.001)	0.08 (0.13)	0.07 (0.19)	−0.06 (0.27)	0.20 (<0.001)

**Table 6:** Binary (stepwise) logistic regression to predict cannabis use in the last year based on family climate and intention to use cannabis, controlling for age and gender.

	B	Wald	*p*	OR	IC 95% OR
Gender	−0.13	0.15	0.70	0.88	0.46:1.69
Age	0.03	0.10	0.76	1.03	0.84:1.27
Subjective Norm	0.85	17.39	<0.001	2.33	1.57:3.48
Intention to use	2.03	43.79	<0.001	7.61	4.17:13.77

B = B coefficient; OR = odds ratio; IC = confidence interval.

**Table 7:** Multiple regression (stepwise) to predict cannabis-use problems (CAST) based on family climate and intention to use cannabis, controlling age and gender.

	B	*t*	*p*
Gender	0.05	1.19	0.24
Age	−0.05	−1.23	0.22
Self-efficacy	0.67	15.46	<0.001
Intention to use	−0.10	−2.23	0.03

## Data Availability

All data are available in this manuscript.
